# Academic attainment in international medical students might be optimised by educating them about cognitive load theory

**DOI:** 10.5116/ijme.6238.4dfd

**Published:** 2022-04-05

**Authors:** William Atiomo, Yvonne Mbaki, Kartikeya Bhardwaj, Pamela Hagan

**Affiliations:** 1 College of Medicine, Mohamed Bin Rashid University (MBRU), Dubai, UAE; 2 School of Medicine, University of Nottingham, Nottingham, UK

**Keywords:** Academic attainment, international medical students, cognitive load theory, human memory, working memory

## To the Editor

As a definition, cognitive load (CL) is the amount of information working memory can hold at any one time. The conceptualisation of CL theory aligns with the framework architecture of human memory, consisting of two key aspects: short-term or working memory and long-term memory. Short-term memory is of finite capacity and duration. It can only hold up to 4-7 chunks of information at any one time, whilst long-term memory is infinite in capacity. During initial learning, the transfer of new information from sensory into working memory takes place and is thereafter processed and transferred into long-term memory for future retrieval when required. Notably, CL theory assumes three components: intrinsic, extraneous, and germane. Intrinsic load is the mental effort required to process new information that is directly relevant to learning. On the other hand, extraneous CL includes distractors, which take up space in working memory (e.g., noise, irrelevant material on PowerPoint slides, negative emotions in the learner such as anxiety) whilst germane CL is the mental effort put into acquisition/development of schemas of information to be held in long term memory. Within the education context, learning optimisation requires a balance of these three components where there is the need to manage intrinsic CL, minimise extraneous CL and promote germane CL

Our interest in CL theory evolved from a reflection on our specific roles as providers of support to international medical students (IMSs) at our institution. Specifically, within this role, we were keen to explore the possible causes of the awarding gap observed between IMSs compared to home students.^1^ Whilst reasons for the awarding gap, like that seen, amongst some students of different ethnicities, are yet to be fully elucidated, a range of factors attributed to potentially increasing extraneous CL have been proposed, specifically to IMSs.^2^^-^^4^ These include the manifestation of negative emotions due to homesickness, social isolation, cultural differences and challenges, the need to learn in a new language and real or perceived racial bias as named examples.

This article describes our pursuit to address part of the causes attributed to the awarding gap by educating IMSs, new to the UK education system, about CL theory, during their induction programme. Notably, although this intervention was UK based, the concepts described here are relevant to all students studying away from their country of permanent residence.

As a collaborative effort between faculty staff and members of the student run medical society, our intervention involved the delivery of an interactive session on CL theory as part of the induction programme to international medical students. As an overview, the objective of this interactive session was to outline potential challenges faced by international medical students with respect to academic attainment and subsequently encourage discussions on mitigating for this by exploring various interventions evidenced within the literature.^5^ Specific to the session’s agenda, the principles of CLT were introduced, explored and a description of how these were linked, including factors which optimise academic achievement in higher education were discussed.^5^

Our exploration of the literature revealed that the top four factors associated with increased academic achievement in students in higher education were student peer-assessment, student’s perceptions of their own academic work performance capability, teacher’s preparation/organisation of the course and teacher’s clarity.^5^ Alongside communicating the aforementioned factors during the session, the concept that “student’s own perceptions of academic performance capability” may reflect high germane CL or a reduction in extraneous CL from emotional self-regulation and motivation was articulated. Moreover, the concept of a link between“teacher-related factors” and the management of intrinsic CL and reduction of extraneous CL (through the presentation/course design) was mentioned.

Whilst the aforementioned set out to highlight positive factors attributed to increasing academic achievement, the top four factors that are known to affect academic attainment negatively were discussed. Specifically, examples such as procrastination, test anxiety, a surface approach to learning, and academic self-handicapping (constructing impediments to performance to protect or enhance one perceived competencies, e.g., claiming illness) were mentioned. Associating these to CL, test anxiety and academic self-handicapping are linked to extraneous CL, as negative emotions are thought to be associated with increased extraneous CL. Although the links between procrastination and a surface approach to learning and CL are less obvious, they could indicate underlying factors linked to negative emotions, a desire for perfectionism, poor engagement, or poor understanding of the teacher’s presentation due to language barriers or poor presentation techniques, both of which relate to extraneous and intrinsic CL respectively. Conversely, a surface approach to learning may result in fewer schemata or patterns being formed to promote germane CL.

As part of the session, experiential practical advice was provided with the objective of minimising extraneous CL to enhance academic attainment potentially. From an academic perspective, this advice included an emphasis on time management with recommendation to commence revision early and often, complemented by engaging with formative assessments. Socially, students were encouraged to embed themselves into the student community, consciously seek out friendship groups, and nurture their sense of belonging. All these aspects proposed were to mitigate against the factors mentioned previously, such as homesickness, cultural challenges and language barriers, as well as developing emotional self-awareness and regulation as a means of acknowledging when to seek help if struggling, e.g., counselling or academic support. Strategies to manage intrinsic CL discussed included the need to seek clarity and understanding following teaching sessions.

In summary, an understanding of how a reduction of factors that contribute to extraneous CL and those that lead to the management of intrinsic and promotion of germane CL and how academic attainment could be influenced is explored with our IMSs early in the transition into medical school to ensure they flourish and attain to their maximal potential.

On reflection, the strengths of our approach were based on early communication of information on practical educational strategies that should improve educational attainment^5^ and conceptualising these factors within an educational theoretical framework (CLT), very early on in the higher education journey of our students. Feedback indicated that our IMSs found these induction sessions useful. In retrospect, hence a limitation to our findings, we could have designed a prospective study to explore if academic attainment improved in our IMSs compared to previous cohorts. As our plan is to execute this intervention again for future cohorts, our reflection is that there appears to be evidence from the literature that theoretically, applying the concepts of CLT might translate into improved academic attainment in IMSs. We would therefore encourage more research into this area from the wider educational research community.

### Conflict of Interest

The authors declare that they have no conflict of interest.
